# Polycystic kidney disease: an unrecognized emerging infectious disease?

**DOI:** 10.3201/eid0302.970204

**Published:** 1997

**Authors:** M A Miller-Hjelle, J T Hjelle, M Jones, W R Mayberry, M A Dombrink-Kurtzman, S W Peterson, D M Nowak, F S Darras

**Affiliations:** Department of Biomedical and Therapeutic Sciences, University of Illinois College of Medicine at Peoria 61656, USA. Marcia.A.Miller@uic.edu

## Abstract

Polycystic kidney disease (PKD) is one of the most common genetic diseases in humans. We contend that it may be an emerging infectious disease and/or microbial toxicosis in a vulnerable human subpopulation. Use of a differential activation protocol for the Limulus amebocyte lysate (LAL) assay showed bacterial endotoxin and fungal (1-->3)-beta-D-glucans in cyst fluids from human kidneys with PKD. Fatty acid analysis of cyst fluid confirmed the presence of 3-hydroxy fatty acids characteristic of endotoxin. Tissue and cyst fluid from three PKD patients were examined for fungal components. Serologic tests showed Fusarium, Aspergillus, and Candida antigens. IgE, but not IgG, reactive with Fusarium and Candida were also detected in cyst fluid. Fungal DNA was detected in kidney tissue and cyst fluid from these three PKD patients, but not in healthy human kidney tissue. We examine the intertwined nature of the actions of endotoxin and fungal components, sphingolipid biology in PKD, the structure of PKD gene products, infections, and integrity of gut function to establish a mechanistic hypothesis for microbial provocation of human cystic disease. Proof of this hypothesis will require identification of the microbes and microbial components involved and multifaceted studies of PKD cell biology.


### Polycystic Kidney Disease: An Unrecognized Emerging Infectious Disease?

**Marcia A. Miller-Hjelle,* J. Thomas Hjelle,* Monica Jones,* William R. Mayberry,† Mary Ann Dombrink-Kurtzman,‡ Stephen W. Peterson,‡ Deborah M. Nowak,* and Frank S. Darras***
*University of Illinois College of Medicine at Peoria, Peoria, Illinois, USA; †East Tennessee State University, Johnson City, Tennessee, USA; and ‡U.S. Department of Agriculture, Agricultural Research Service, Peoria, Illinois, USA

Polycystic kidney disease (PKD) is one of the most common genetic diseases in humans. We contend that it may be an emerging infectious disease and/or microbial toxicosis in a vulnerable human subpopulation. Use of a differential activation protocol for the Limulus amebocyte lysate (LAL) assay showed bacterial endotoxin and fungal (1→3)-β-D-glucans in cyst fluids from human kidneys with PKD. Fatty acid analysis of cyst fluid confirmed the presence of 3-hydroxy fatty acids characteristic of endotoxin. Tissue and cyst fluid from three PKD patients were examined for fungal components. Serologic tests showed *Fusarium*, *Aspergillus,* and *Candida* antigens. IgE, but not IgG, reactive with *Fusarium* and *Candida* were also detected in cyst fluid. Fungal DNA was detected in kidney tissue and cyst fluid from these three PKD patients, but not in healthy human kidney tissue. We examine the intertwined nature of the actions of endotoxin and fungal components, sphingolipid biology in PKD, the structure of PKD gene products, infections, and integrity of gut function to establish a mechanistic hypothesis for microbial provocation of human cystic disease. Proof of this hypothesis will require identification of the microbes and microbial components involved and multifaceted studies of PKD cell biology.

Examining the hypothesis that polycystic kidney disease (PKD) is an emerging infectious disease and/or microbial toxicosis in a vulnerable population of humans must begin with a review of the conceptual tools that relate disease etiology and progression to the identification of microbes, their cellular components, and shed toxins in affected persons (1). An emerging infectious disease can be defined, in part, as an existing disease for which microbes are newly recognized as a causative factor and/or as a factor contributing to disease progression. The microbe may be 1) present at the site of a lesion, 2) disseminated throughout the body, or 3) localized to an anatomic site separate from the primary lesions. In this third case, one or more toxins released by the microbe into distribution fluids, usually blood, produce the pathologic effect(s); this process resembles microbial toxicosis, where the patient is exposed only to the toxin(s) present in the diet, environment, and gut microflora. Distinguishing between an infectious disease and microbial toxicosis is essential if the source of the toxin is to be removed or reduced. Concurrent infection and microbial toxicosis (e.g., endotoxicosis, mycotoxicosis) can also occur. Although mycotoxicosis is commonly understood to refer to the absorption of small organic fungal toxins, such as aflatoxin, fumonisin, or trichothecene, detection of toxin in body fluids and tissues is often difficult. Exposure to toxin may be episodic. For many known and newly discovered microbial toxins, analytical methods pertinent to the levels of toxins involved in acute and chronic disease, especially in vulnerable subpopulations, need to be developed and verified in human tissues and fluid. We have taken the view that the presence of

Address for correspondence: Marcia A. Miller-Hjelle, Department of Biomedical and Therapeutic Sciences, University of Illinois College of Medicine at Peoria, P.O. Box 1649, Peoria, IL 61656; fax 309-671-8403; e-mail: Marcia.A.Miller@uic.edu.

signature components of microbial genera/species that produce toxins may indicate that all components of the microbe, including its shed toxins, are also present. As a corollary, absorption of the detectable microbial components, which are often larger in molecular size than the classical mycotoxins and endotoxins, should presume the absorption of other components and toxins from this organism.

On its face, PKD would not appear to be an emerging infectious disease. Classically viewed as an inherited disease that follows Mendelian genetics, PKD in its autosomal dominant form affects 600,000 people in the United States at a rate of 1 in 400-1,000 persons; the autosomal recessive form occurs in 1 per 44,000 births. An acquired form of PKD occurs in patients on renal dialysis. Animal models of inherited and chemically induced PKD have been described (2-4). The current consensus is that fundamental anomalies in cell differentiation or maturation explain the array of anomalies in cystic renal epithelium. Although cysts can sometimes be detected in utero, loss of kidney function to the point of end-stage renal disease occurs by the sixth decade in 50% of autosomal-dominant PKD patients. Despite substantial progress in molecular genetics studies of PKD, the role of defective gene products in its causation and progression to end-stage renal disease has remained elusive (3,5,6). Indeed, additional factors have been proposed as being necessary for progression of PKD to end-stage renal disease. PKD patients have higher rates of renal and urinary tract infections and higher rates of illness and death from infection in general than healthy persons (7,8). Since these infections in otherwise-healthy-persons do not lead to PKD, PKD patients could be exhibiting heightened vulnerability to infection and sensitivity to microbial cell components and toxins. How such sensitivity and vulnerability might occur is unknown. The disease does not appear to involve an immunocompromised state. Thus, alternative possibilities need to be considered, such as altered colonization of the uroepithelium and altered bowel structure and colonization.

### Microbes and Microbial Components

Werder et al. (9) performed perhaps the critical experiment in establishing a pathogenic role for microbes in PKD. Using genetically cystic mice, they found that mice grown under germ-free conditions survived substantially longer than cystic littermates grown under ambient conditions. Gardner et al. (10,11) extended these observations when they reported that rats fed a chemical cystogen also had lower rates of renal cystogenesis when raised under germ-free conditions; moreover, coadministering the ubiquitous cell wall component of gram-negative bacteria, lipopolysaccharides (LPS), and the chemical cystogen produced higher rates of cyst formation than using cystogen alone. Thus, LPS was considered a provocateur of the underlying genetic (or chemically induced) susceptibility to renal cystogenesis. From this line of research has emerged the possibility that LPS, either alone or in combination with other microbial or environmental factors, promote disease progression, and removal of such provocateurs would slow the progression of PKD to end-state renal disease.

Endotoxin is the mixture of LPS and other bacterial cell wall proteins and constituents that is found in bacterial infections (12). LPS is isolated by solvent extraction of bacterial cell walls; its chemical purity may vary across preparations. The portion of LPS responsible for most of its known biological activities is Lipid A; the carbohydrate side chains also elicit responses in humans (13). The chemical composition of LPS depends on the genera and species of bacteria from which it is isolated (13,14). As knowledge of the structure and biological activities of LPS has increased and merged with the infectious disease vocabulary based on endotoxin, these terms are sometimes used interchangeably. For example, the highly purified LPS used as positive reference material in the Limulus amebocyte lysate (LAL) assay is called Control Standard Endotoxin. Even though interchanging the terms LPS and endotoxin may promote appreciation of microbial involvement in disease, we prefer the term endotoxin in discussing disease because it respects the structural and pharmacologic heterogeneity of LPS and mixtures of other microbial components present in vivo.

When Miller et al. (15) measured putative endotoxin levels by the classic LAL assay in culture-negative urine samples of male PKD patients with no clinically apparent infection, 80% contained detectable LAL-positive material, while the urine samples of healthy male volunteers did not. In cyst fluid from PKD patients, Gardner et al. (16) reported LAL-positive material and cytokines typically induced by LPS. The origins of the renal and urinary endotoxin were not known; possible sources include the gut microflora, occult urogenital infections, cryptic colonizations, and abnormal handling by the liver of the normal daily load of absorbed endotoxin. Enhanced exposure to endotoxin in the PKD patient is important since endotoxin plays a fundamental role in human health and disease. In low doses, endotoxin enhances the immune system, but in larger amounts it is lethal. In combination with drugs and other substances, it can cause tissue damage and severe disease; examples of endotoxin synergy with other substances resulting in disease include alcohol in cirrhosis, aspirin in Reyes syndrome, and trichothecene T-2 toxin (a mycotoxin from *Fusarium*) in gastrointestinal tract damage (17). Endotoxin can also promote the translocation of bacteria from the gut into the blood (18). In susceptible persons, chronic exposure to endotoxin has been linked to rheumatoid arthritis (19); tentative links with atherosclerosis have also been reported (20). Although suggestive of endotoxin in PKD, the LAL methods used above were not specific for endotoxin, since they were susceptible to false positive and/or negative results.

### Bacterial Endotoxin in Human PKD Renal Cysts

In this report, we describe our recent findings concerning 1) endotoxin levels recorded after recent improvements in LAL methods, 2) analysis of fatty acids specific for bacterial endotoxin, and 3) the presence of other microbial components in human cyst fluid. The LAL assay (Figure 1) is the classic method used to detect the low levels of endotoxin in biologic fluids and pharmaceuticals.

Both endotoxin (in eight of eight patients) and (1→3)-β-D-glucans (in two of eight patients) were detected when methods based on differential activation of the two Limulus pathways were applied to cyst fluids from six autosomal-dominant PKD patients, one autosomal-recessive PKD patient, and a patient with simple cysts, where 200 ml from a single simple cyst were recovered (Table 1). None of these patients exhibited clinical evidence of infection, had been on hemodialysis, or received human immunologic products before nephrectomy, which could have accounted for ß-D-glucan reactivity (26). For six patients (four autosomal dominant, one autosomal recessive, and one with simple cysts), only endotoxin was detected in cyst fluids; gel clot end points for both the LAL and LAL+laminarin assays were equal

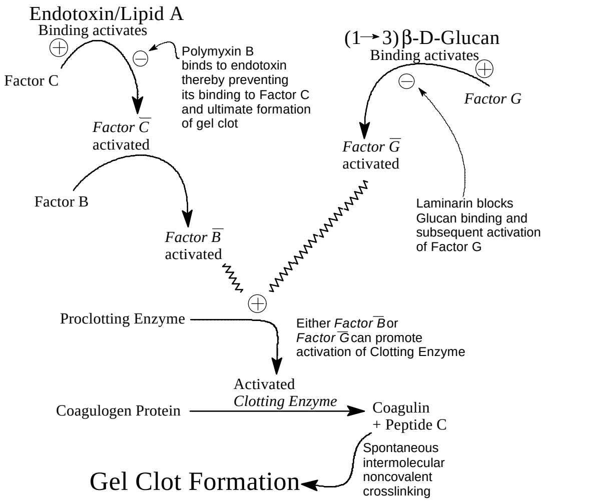

Figure 1. Cascade of biochemical interactions and reactions leading to gel clot formation in the Limulus amebocyte lysate assay. Endotoxin binding to Factor C via the Lipid A moiety results in its activation and in turn the sequential activation of Factor B, which results in the subsequent activation of the proclotting enzyme. Binding of polymyxin B to endotoxin blocks Limulus reactivity in those samples where endotoxin is the initiating molecule. In contrast, (1→3)-β-D-glucans bind to a different component, Factor G, in the Limulus assay reagent, thereby leading to its activation and subsequent cascade of reactions to gel formation. The addition of laminarin, an inhibitor of glucan binding to Factor G, to Limulus assay reagent blocks reactivity of samples containing glucan (21).

Lipid A binds to and activates Factor C resulting in a cascade of enzymatic reactions leading to gel clot formation, the positive endpoint (22). Also present in the classic LAL reagents is Factor G, which is responsible for a side cascade (i.e., the alternative pathway). Factor G is stimulated by (1→3)-ß-D-glucans, associated primarily with fungal cell walls (23). Addition of laminarin, an inhibitor of glucan binding to Factor G (24), permits a more specific measurement of endotoxin-induced gel clot formation and provides a means of screening for (1→3)-β-D-glucans in biological samples, when used in a differential activation protocol with other inhibitors and activators. As two internal controls, polymyxin B can block the LAL reactivity of endotoxin (25), and known quantities of Control Standard Endotoxin can be added to duplicate samples to detect inhibition of the assay.

for each fluid tested, indicating that the β-D-glucan stimulated pathway was not activated. When exposed to polymyxin B, LAL-positive cyst fluids became negative. This is consistent with the conformational changes that occur when this antibiotic binds to Lipid A to form a stoichiometric

Table 1. Limulus assay results from human kidney cyst fluids

| Donor No. | Patient data | No. Cysts Tested | Cysts ET positive (%): Endotoxin units/ml[a] | Cysts ß-D-glucan positive (%): pg/ml |
|---|---|---|---|---|
| 1 | AD[b] 45 yr F | 5 | 3 (60%): 0.48 EU/ml | nd[c] |
| 2 | AD 52 yr F | 6 | 4 (67%): 1.71 EU/ml | nd |
| 3 | AD 48 yr M | L[d] 9 | 5 (56%): 1.45 EU/ml | nd |
| | | R[e] 11 | 6 (55%): 2.27 EU/ml | nd |
| 4 | AD 41 yr F | 16 | 3 (19%): 0.88 EU/ml | 3 (19%): 120 pg/ml |
| 5 | AD 37 yr M | 11 | 2 (18%): 3.84 EU/ml | 6 (55%): 106 pg/ml |
| 6 | PD[f] AD 43 yr F | 13 | 4 (31%): 0.26 EU/ml | nd |
| 7 | AR[g] 5 wk M | pool(8) | 3.84 EU/ml | nd |
| 8 | SC[h] 48 yr F | 1(200ml) | 3.84 EU/ml | nd |

[a]Mean EU/ml or pg (1→3)-β-D-glucan/ml
[b]Autosomal-dominant polycystic kidney disease (PKD)
[c]Not detected
[d]Left kidney
[e]Right kidney
[f]Peritoneal dialysis
[g]Autosomal recessive PKD
[h]Simple kidney cyst (not PKD)

Kidneys were from seven patients who had received or were awaiting renal allografts. Cyst fluid was obtained ex vivo from both surface (cortex) and sub-surface (medullary) cysts by aseptic aspiration from seven excised kidneys (post-nephrectomy) of PKD patients; simple cyst fluid was recovered by radiologically guided needle aspiration. All kidneys and cyst fluids were collected under an approved Institutional Review Board protocol. The mean number of cysts aspirated per kidney was 10 (range 1-21); two kidneys were obtained from one of the patients with both being used for studies. Fluid from individual cysts in each kidney were collected separately, except for the autosomal-recessive PKD patient in whom cyst volumes were too small (~ 0.1 ml) for individual testing. Therefore, fluid from eight cysts in close proximity were pooled. All procedures used pyrogen-free materials in the collection, handling, and processing of kidneys and cyst fluids and preparation of commodities and reagents for the Limulus assay. Depyrogenation was carried out at 250°C for 4 hours, which eliminates both endotoxin and (1→3)-ß-D glucans (27). Cyst fluids were stored at -70°C. Assessment parameters of cysts included color and size; for fluids, color, turbidity, pH, mg protein/ml, specific gravity, and blood. A commercial dipstick was used to detect and semiquantitate cyst fluid pH, specific gravity, and blood (Ames 10 SG Multistix, Miles, Inc., Elkhart, IN, USA). Blood sensitivity was 0.015-0.062 mg/dL hemoglobin, equivalent to 5-20 intact red blood cells per microliter. Results were recorded as 1-4+. For specific gravity, determinations permitted ranged from 1.000-1.030; for pH, 5.0-9.0. Protein was by the method of Lowry et al. (28).

The LAL gel clot end point assay (22) was performed according to the manufacturer's instructions (Charles River ENDOSAFE, Charleston, SC). The mechanism of the LAL assay is illustrated in Figure 1. To quantify endotoxin, undiluted samples and serial twofold dilutions through 1:64 were tested. To detect false positives due to activation of the alternate (1→3)-β-DG pathway, LAL assay reagent fortified with the glucan inhibitor laminarin was used (23). Laminarin was courtesy of Dr. J. Cooper, Charles River ENDOSAFE, Charleston, SC. Laminarin had been rendered endotoxin free by treatment with 0.2 N NaOH at 50°-60°C for 6 hours; treatment did not affect activity of laminarin in the LAL assay (23). The standard gel clot end point method was used, except laminarin was added to the LAL assay reagent (23). Minimum sensitivity of both assays was 0.03-0.06 Endotoxin Units (EU)/ml (3-6 pg control standard endotoxin, CSE; 10 EU/ng of EC-5 (*E.coli* O133) U.S. Standard endotoxin*;* for β-DG, $^{3}$10 pg/ml (29).

Known endotoxin-positive (tap water) and -negative (pyrogen-free water) samples were run as controls along with a CSE curve. The endotoxin concentration was estimated by multiplying the maximum dilution producing a positive test against the sensitivity of lysate that was verified daily. The content of β-DG in cyst fluid was estimated by the difference in titers between the endotoxin specific assay (LAL+laminarin) and the conventional LAL assay (30). Portions of all samples were additionally spiked with 0.25 EU CSE and tested for inhibitory substances. If inhibition was observed in the LAL assays, samples were boiled for 5 minutes, cooled to room temperature, and retested. Samples were incubated, in some cases after boiling, for 30 min at room temperature with polymyxin B (5 mg/assay) before LAL testing. Polymyxin B inhibits the LPS-activated pathway via Factor C by its direct binding to LPS, which yields a nonreactive complex (25). An alternative approach was to use 20% dimethyl sulfoxide (DMSO) to inhibit Factor C directly (23); DMSO was not used to permit detection of nonendotoxin, but LAL-reactive materials that were not inactivated by binding to polymyxin B. However, in our system DMSO did completely block the LAL reaction in the presence of CSE.

After centrifugation of the cyst fluid, the LAL-positive material was located only in the supernate for autosomal-recessive- and simple cyst-derived fluids. For autosomal dominant-derived fluids, the reactive material was located in the pellet. Thus, centrifugation of fluids to remove particulate material before the LAL assay may influence results.

LAL assay inhibition was detected in nearly two-thirds of 73 cyst fluids tested, including autosomal recessive and simple cyst fluids. Dilution of cyst fluid through 1:16 was often required to overcome the inhibitory effect in unboiled specimens. Boiling of entire fluids or supernates and pellets for 5 minutes eliminated LAL assay inhibition in virtually all specimens at all dilutions. Exceptions were some undiluted and 1:2 dilutions of chocolate-colored cyst fluids, where inhibition was not eliminated in both test and spiked specimens of autosomal dominant fluids.

complex that is inactive in the assay (25). In contrast, kidney tissue from one male patient (donor 4) and one female patient (donor 5) yielded cyst fluids that contained separately endotoxin or β-D-glucan; however, no single fluid contained both substances (Table 1). Fluids that were LAL positive in the presence or absence of polymyxin B, but negative in the presence of laminarin are consistent with the detection of β-D-glucan. Donor 6 had been on peritoneal dialysis for slightly more than 1 year before nephrectomy. Although cyst fluids were all initially LAL negative, when the incubation time of the LAL assay was increased from 1 to 2 hours, 4 of 13 cyst fluids were endotoxin positive, albeit at much lower concentrations than the fluids from patients not on dialysis. None of the cyst fluids from donor 6 were positive for β-D-glucan after longer incubations. Longer incubation times might also have produced LAL-positive material in LAL-negative cyst fluids from the other donors; therefore, we view the percentage of LAL-positive cyst fluids for each patient as a minimum value.

The concentrations of endotoxin-specific material in autosomal dominant cyst fluids was 0.12 to 3.84 EU/ml, with substantial levels of endotoxin (3.84 EU/ml) also found in a pool of eight cysts from one autosomal recessive kidney and simple cyst fluid from one donor. The mean concentration of endotoxin across all of the autosomal dominant fluids tested (including those that were LAL negative) was 0.65 EU/ml. Using 10 EU/ng as a conversion factor yields 65 pg endotoxin/ml cyst fluid; normal plasma levels of endotoxin are <4-5 pg/ml (31). Intravenous injection of 5 EU/kg body weight (350 EU/70 kg) can induce shock in the average adult male (21). We estimate that a single cystic kidney in an adult male (3-5 kg kidney weight of which 33% is cyst fluid) contains 648 to 1,080 EU/kidney or about two lethal doses of endotoxin per kidney. Unexplained fever and flank pain in PKD patients have been attributed to release of IL-1 from rupture or hemorrhage of cysts (27). On the bases of the high levels of endotoxin observed in this study and the high sensitivity of humans to endotoxin, we propose that the release of endotoxin from the cyst into the peritoneum or blood may be an important initiator of a cascade of biologic events after leakage or rupture of renal cysts.

For one donor (donor 3) both kidneys were available for cyst fluid analysis (nine left and 11 right kidney cysts, respectively). When the frequency of LAL-positive fluids (endotoxin) was compared, no significant difference was found (Table 1). However, a threefold difference in the frequency of inhibitor detection was observed in left kidney cyst fluids compared with the right kidney (90% vs. 31%, respectively). Thus, LAL testing of fluids for endotoxin requires vigilance for both false negative and positive materials.

### Signature Bacterial Fatty Acids

If endotoxin is present in renal cysts, fatty acids unique to endotoxin can be used to confirm its presence. The Lipid A region of LPS contains on average four 3-hydroxy (3-OH) acyl groups of various chain lengths (13,14). Acid hydrolysis of LPS releases the 3-OH fatty acids and other fatty acids. Diverse bacterial genera exhibit characteristic ratios of fatty acids of various chain lengths and patterns of hydroxylation; compilations of such signature fatty acid profiles are available for many genera (14). Initially, aliquots of five cyst fluids were analyzed in a single blind manner by gas chromatography-mass spectrometry as described by Mayberry and Lane (32). 3-Hydroxy (3-OH) fatty acids of *n*C:12:0 and *n*C:14:0 carbon length were detected but not quantified in the three cyst fluids that were positive in the endotoxin specific LAL assay; they were not detected in the two cyst fluids that were LAL negative. Thus, complete concordance of LAL reactivity and signature fatty acid analysis was observed. 3-OH fatty acids (C:14, C:16) were also reported in LAL-positive cyst fluids by a separate reference laboratory (Microbiological Insights, Inc., Knoxville, TN). Additional chemical analyses of cyst fluid are required before a classic signature fatty acid profile and linkage to one or more bacterial genera are possible. Also, we cannot discount the possibility that 3-OH fatty acids released during degradation of endotoxin were incorporated into mammalian sphingolipids that were positive in the LAL assay, thereby accounting for both the presence of such fatty acids and LAL reactivity in cyst fluids.

### (1→3)-β-D-Glucans (β-DG)

In addition to endotoxin, (1→3)-β-D-glucans were identified in cyst fluids from two patients by differential activation of the LAL assay (Table 1). The range of β-DG reactive material in cyst fluids was estimated at 40 to 160 pg/ml. In plasma of healthy persons, levels of β-DG are lower than 6.9 pg/ml (26). β-DG, a ubiquitous constituent of filamentous fungal and yeast cell walls (33), is exceeded only by endotoxin in its reactivity in the LAL assay; glucans lacking a 1→3 linkage are not LAL reactive (34), nor are mannans, dextrans, and cellulose (24,26). Although diverse forms of glucans are components of fungal cell walls, those with (1→3)-β linkages are the most potent in causing hypersensitivity pneumonitis and other severe pulmonary illnesses (35). Although β-DGs are indicative of fungal cell walls, their occurrence alone does not identify the genera and species of the fungus that produced it. To confirm the presence of fungal components and determine the source of β-DG, serologic tests were performed on three β-DG positive and two β-DG negative cyst fluids from three autosomal-dominant PKD patients (Table 2). Cyst fluid from donor 6 that was negative for LAL-reactive material was also free of detectable fungal antigens and anti- from both kidneys containing β-DG-positive material (donors 4 and 5; Table 1) were also positive for fungal antigens. Fluid from donor 5 also exhibited fungal antibodies; fluid from donor 4 was not tested for antibodies to fungi.

Fungal serologic tests provided insights into potential sources of β-DG. Initial measurements showed *Fusarium solani* antigen. These findings suggest the antigen to be from *F. solani* or a shared antigen present in other *Fusarium* species (L. Kaufman, pers. comm.). IgE (but not IgG) antibody to *Fusarium moniliforme* was also detected. *Aspergillus* galactomannan antigen and *Candida albicans* serotype *A. mannan* were present in selected cyst fluids. Cross-reactivity of *Fusarium* with *Aspergillus* and *Penicillium* is unlikely as 1) the *Fusarium* antibody (prepared against the mycelium) was cross-absorbed with *Aspergillus*, and 2) the immunodominant group of *Aspergillus* and *Penicillium* species, galactomannan with a galactofuranosyl moiety (38), is absent in *Fusarium* species (39). As the serologic tests for *Fusarium* and *Aspergillus* in cyst fluids are experimental and have not been evaluated clinically, these data are indirect presumptive indication of the presence of these fungal antigens. The paucity of fungal serologic and chemical methods for detection and identification of emerging fungal pathogens in human tissues and body fluids and fungal components in the human diet has been noted by others.

Also detected were IgE antibodies to *Candida*. IgE antibodies to both *Fusarium* and *Candida* in cyst fluid suggest recruitment of immunologic defenses against fungi. The site of production of these IgEs and route of entry into

Table 2. Serologic results of five cyst fluids from three autosomal-dominant PKD patients tested for fungal antigens or antibodies to fungal components

| Donor;Cyst No. | LAL[a] Findings | Serologic Findings |
|---|---|---|
| D6;1 | Neg. Et[b];Neg. ß-DG[c] | Antigens not detected |
| D6;2 | Neg. ET;Neg. ß-DG | IgE and IgG reactive with fungi were not detected |
| D4;3 | Neg. ET;Pos. ß-DG | Antigens: *Fusarium solani* at 1:2 dilution; *Aspergillus* galactomannan at >50 ng/ml |
| D5;4 | Neg. ET;Pos. ß-DG | Antigens: *Aspergillus* galactomannan at >50 ng/ml; *Candida albicans* serotype A mannan at 10 ng/ml |
| D5;5 | Neg. ET;Pos. ß-DG | IgE reactive with *Fusarium moniliforme*; *C. albicans* (weak positive) |

[a]Limulus amebocyte lysate assay
[b]Endotoxin
[c]ß-D-glucan

Fungal serology was performed on three cyst fluids from donors 4 and 5; (Table 1) with ß-DG positivity and two cyst fluids from donor 6, which were negative for both endotoxin and ß-DG after extended incubation in the LAL assay. Fluids were tested for antibody to *Penicillium notatum* (*Penicillium chrysogenum*) and *Penicillium frequentans*, *Aspergillus fumigatus*, *Candida albicans* (yeast), and *Fusarium moniliforme*. The Pharmacia CAP systems RAST FEIA (IgE) and IgG FEIA were employed (Pharmacia & Upjohn Diagnostics, Kalamazoo, MI, USA). The tests were modified by substituting cyst fluid for plasma; interference of fluid with the test was not observed. Tests to detect fungal antigens were performed by the Centers for Disease Control and Prevention, Atlanta, GA. To detect *Fusarium* sp. antigen, a modified aspergillosis microimmunodiffusion test (36) was used with experimental rabbit anti-*Fusarium solani* antibody; kidney cyst fluid was substituted for plasma. A double-antibody sandwich enzyme immunoassay was used to detect *C. albicans* serotype A mannan concentrations in cyst fluids; specificity of this test in sera is reported as 100% (37). An experimental ELISA inhibition assay was used to detect *Aspergillus* galactomannan (Steven Hurst, unpub. protocol) Standard serum reference curves were used for extrapolating quantities of *C. albicans* mannan and *Aspergillus* galactomannan in cyst fluids. However, these tests were not designed for cyst fluids, but for serum antigen detection, and must, therefore, be viewed as estimated quantities.

the cyst are unknown; a hypersensitivity reaction cannot be discounted. In human and experimental PKD, the severity of cystic disease correlates with the numbers of circulating neutrophils; neutrophilia is related to exposure to endotoxin and cytokines, both of which have been reported in cyst fluids (16). Our finding of fungal antigens and antibodies raises the additional possibility of leukocyte activation in PKD by ß-DG and mannan released from fungi, as ß-DG and mannan have been reported to stimulate cytokine production (40).

Serologic testing is frequently used to detect fungal infection. Cyst fluids collected from PKD patients at sequential times during progressive stages of the disease were not feasible. Therefore, quantitative and/or qualitative changes in antigen or antibody titers could not be used to determine active fungal infections in these patients. Although antigens of *Aspergillus* or *Fusarium* species are generally interpreted as apparent or covert infection, another possibility is the absorption and distribution of fungal components independent of infection (i.e., mycotoxicosis versus infection). While *Aspergillus* enters primarily by inhalation, this route does not appear to be the predominant source of *Fusarium*; fewer than 1% of air samples contained *Fusarium* species (41). Fumonisin from *Fusarium* is present in grains, rice, and corn consumed by humans (42), with regional variations in levels and the type and processing of dietary product. Because of their occurrence in the food supply and their effect on experimental animals, it has been proposed that fumonisins may contribute to kidney disease in humans (43).

ß-D-Glucans and mannans are shed by fungi during growth (26,39) and thus, like bacterial endotoxin, can potentially be distributed through blood and lymph. Diverticular disease and other anomalies in the structural and functional integrity of the gut may occur in up to 80% of PKD patients (2,44). In addition to diminished barriers to the absorption of gut-derived endotoxin, the outpouchings (diverticula) become overgrown by minor subpopulations of gut microflora (45), thereby generating increased quantities of unusual mixtures of potentially absorbable microbial components. Diverticular disease (44) and acquired renal cystic disease (46) occur in patients on hemo- and peritoneal dialysis. To complete the symmetry of this line of reasoning, outpouchings of the renal tubule in PKD are described as both cysts and diverticula (2). Direct measurement of intracyst pressure and elasticity of the basal lamina have led to the rejection of the obstruction hypothesis (i.e., a physical balloon inflation) of cystogenesis in favor of a cell-mediated restructuring of the basal lamina coupled with electrolyte transport into the cyst to expand intracyst volume (47). Although diverticular disease is thought to be a pressure-driven lesion, gut diverticula in PKD and dialysis patients may also involve a cellular lesion further promoted by pressure. Thus, diverticula in kidney and gut are associated with microbial components. It is not known if the processes of renal cystogenesis, formation of gut diverticula, and the walling off of infecting material are all facets of the same defense response in humans. Endotoxin in relatively high concentrations was found in fluid from a simple cystic kidney (Table 1); intestinal or biliary obstruction and urinary tract infections have been associated with simple kidney cysts (2). Although endotoxin has been proposed as being associated with “high sodium cyst fluids” (47), we observed endotoxin in cyst fluids of low and high sodium content (data not shown).

Relevant to our study is the report of the capacity of ß-DG to prime cell systems, which results in sensitization to bacterial endotoxin and infection (48), an example of ß-DG-endotoxin enhancement; ß-DG and endotoxin also demonstrate a strong synergistic effect on macrophages (49). In addition, there have been reports of synergy between mycotoxin and endotoxin (50).

### Detection of Fungal DNA

To identify fungal DNA as evidence of past or current fungal infection and/or the absorption and distribution of fungal components to kidney cysts, polymerase chain reaction (PCR) methods were used to amplify and thus detect fungal DNA in PKD cyst fluids and kidney tissue. Six cyst fluids from three patients and two samples of kidney tissue from two autosomal-dominant PKD patients were tested; all were positive for fungal DNA. A sample of normal human kidney was negative for fungal DNA (Figure 2 A-C). Because culture confirmation was not achieved from the cyst fluids, expanded studies with species-specific probes are warranted.

Despite substantial effort, we have not been able to culture either bacteria or fungi from PKD cyst fluids by axenic methods, but fungi were detected in cultured epithelial cells from PKD kidney tissue (see below); methods appropriate to culture L-forms or other cell-wall-defective microbes were not performed. Gram stains of cyst fluid supernates and pellets did not show intact bacteria. Electron microscopy also did not show intact bacteria, but occasionally, a field consistent with fungal cell walls was observed (not shown); intact fungi were not observed. Nonetheless, it was only from PKD kidneys where b-DG was identified in cyst fluid that fungal organisms were frequently isolated from PKD epithelial cell cultures. Other human and animal cells isolated and propagated in the laboratory by the same reagents and work spaces were free of fungal growth. The fungi were identified as belonging to the genera *Paecilomyces* and a new species of *Penicillium* (manuscript in preparation). Fungi have been reported, albeit infrequently, as etiologic agents in renal infections in PKD (8). Given all of the above, it is possible that viable fungi were present in the PKD renal tissue, but only

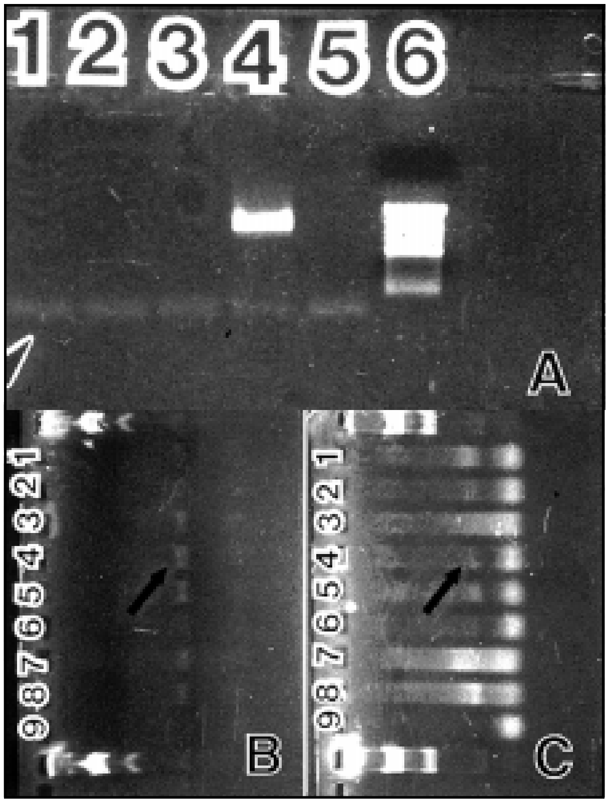

Figure 2. Amplification results of normal and PKD kidney tissue and cyst fluids with universal fungal primers ITS 1 and NL 4. 2A: DNA from healthy human kidney tissue diluted 1:10, 1:100 and 1:1,000 (lanes 1-3); control fungal DNA, *A. tamarii* (lane 4); negative control (lane 5); 1 kb ladder (lane 6); arrow indicates migration front. 2B (NL 4) and 2C (ITS 1): two cyst fluids, donor 6, negative for detectable endotoxin and b-DG (lanes 1 and 5); two cyst fluids, donor 4, positive for b-DG (lanes 2 and 3) and positive for *Fusarium solani* antigen (lane 3); two cyst fluids, donor 5, positive for b-DG (lanes 4 and 6); two PKD kidney tissues, donor 5 (lane 7) and donor 4 (lane 8); negative control (lane 9). Large arrows in 2B and 2C point to 560 bp molecular weight marker; small arrows point to product bands.

**Polymerase Chain Reaction**

Specimens for PCR included 1) normal human kidney tissue (0.46 g) from a 7-year-old girl who died of a head injury; the kidney was not transplantable because of the presence of a hematoma but was otherwise normal; the section used for PCR excluded the area of hematoma. 2) 0.5 g of cystic tissue, devoid of cyst fluid, from each of two autosomal-dominant PKD patients (donors 4 and 5, Table 1). 3) multiple cyst fluids (0.5-1.0 ml each; donors 4, 5, and 6). The positive control consisted of DNA extracted from *A. tamarii* (NRRL 26010) plus primer. The CTAB extraction method for fungal genomic DNA was employed. The universal oligonucleotide primer pair ITS 1 and NL4 for fungi, as previously described (51), was used; NL 4 is the reverse primer. PCR master mix (37) with 1.5 mM $MgCl_2$ and primers in a final reaction volume of 100 ml was subjected to the following thermal cycler parameters (Perkin Elmer Thermo Cycler 1): 96°C—30 sec; 53°C—30 sec and 72°C—30 sec for 40 cycles, followed by a final 7 min extension at 72°C and soak at 4°C. The presence of the expected molecular weight PCR products (600 bp) was confirmed by ethidium bromide staining after separation on a 1.0 % agarose gel. The amplified products were compared visually for similarity on the basis of the presence or absence of bands; variations in band intensity were not considered.

A-C represent the banding patterns obtained by agarose gel electrophoresis of healthy kidney tissue, cystic kidney tissues, and cyst fluids following PCR amplification with the universal fungal primer pair, ITS 1 and NL 4. Amplified fungal products were not detected in the negative PCR controls (2A, lane 5; 2B and C, lanes 9) or normal human kidney tissue (2A, lanes 1-3). In marked contrast, distinct bands of the predicted size of each fungal primer used ( ~ 600 bp; 45) were detected in all cystic kidney tissues and fluids examined from the three patients (2B-C); minor fragments were not detected. The migration patterns were consistent with the positive fungal DNA control (2A, lane 4).

Although fungal DNA might have been anticipated in tissues and fluids from PKD kidneys showing b-DG or positive serologic findings, fungal DNA was also found in PKD cyst fluid from donor 6 (2B and C: Lanes 1 and 5), which lacked these components. The b-DG positive cyst fluids tested from donors 4 and 5 are in Lanes 2 and 3 and 4 and 6, respectively. The fluid depicted in Lane 3 also had positive serologic results for *F. solani* antigen; serologic testing was not done on the other fluids assayed by PCR. Each cyst fluid must be considered an independent sample, requiring direct measurement; assumption of cyst content on the basis of other fluids within the same kidney is not valid (16). Fungal DNA was also detected in kidney tissue from donors 4 and 5 (Figure 2B and C; Lanes 8 and 7). Differences were noted between the two primers used; ITS 1 yielded amplified products in all PKD samples while NL 4 amplified less well, being more concordant with the detection of b-DG. The amplicons are presumptive evidence of fungal DNA.

their remnants were in the cyst fluid. Emerging human awareness of infectious disease may well describe our experience in this study of PKD.

### Infectious Disease, Microbial Toxicosis, and Sphingolipid Biology

Emerging knowledge of sphingolipid biology and PKD's vulnerability to microbial toxins offer an opportunity for a fresh look at PKD. As Spiegel and Merrill (52) have noted, sphingolipids fall into two broad categories, both of which are altered in PKD (53,54). Complex sphingolipids (i.e., ceramide with a carbohydrate or phosphocholine head group on carbon-1) interact with growth factor receptors and the extracellular matrix and adjacent cells and act as binding sites for gram-positive and -negative bacteria and microbial toxins. In the second category of sphingolipids, ceramide sphingoid bases (i.e., sphingosine, sphinganine, phytosphingosine) and their 1-phosphates modify the activity of protein kinases and phosphatases, ion transporters, and a growing number of signal transduction processes. However, the dynamics of human sphingolipid biology occur within the context of a microbe-dominated environment.

Thus, we propose a third category called "microsphingoids," sphingolipid-like molecules of microbial origin that mimic or antagonize the actions or metabolism of human sphingolipids. Examples include, but are not limited to, various mycotoxins (42,55-57), bacterial sphingolipids (58), and endotoxin (59,60). In this report, we have shown the presence of endotoxin and fungal antigens or antibodies to at least *Fusarium*, *Aspergillus*, and *Candida* in human PKD tissues and fluids; from human PKD cells in vitro we have encountered *Paecilomyces* and *Penicillium*. These fungi produce mycotoxins that inhibit multiple enzymes required for sphingolipid biosynthesis (55; Figure 3) and alter the activity of various elements of signal transduction cascades, such as protein phosphatases, calmodulin, and GTP-binding proteins (56,61). Such mycotoxins are also found in the human diet, which is itself an emerging concern in food safety (42,43,55). Rietschel et al. (13) noted the similarity of LPS to glycosphingolipids. Wright and Kolesnick (59) have reviewed the structural similarity of the Lipid A region of bacterial LPS with ceramide and the ability of LPS to mimic the ceramide-induced alterations in protein kinase and phosphatase activities. Endotoxin is also reported to alter the structure of mammalian sphingolipids (62) and to initiate cytokine-mediated cascades that generate ceramide, sphingosine-1-phosphate, and lysosphingolipids, all of which exhibit biologic activity (13,63). Not to be ignored are bacterial sphingolipids formed by genera such as *Bacteriodes*, which represent approximately 30% of the gut microflora (58). The role of bacterial sphingolipids and their metabolites in human biology and their bioavailability in disease are poorly understood. The term "microsphingoid" is intended to highlight the contribution of microbes to sphingolipid biology in human health and disease.

Could pertubations in sphingolipid biology caused by genetic anomalies and/or "microsphingoids" account for both infantile autosomal recessive and adult onset forms of kidney cystic disease? Hannun (64) has reviewed the role of sphingolipids as "biostats" that regulate diverse cellular programs executed in response to various stimuli. Such biostatic regulation could account for both acute and chronic changes in cellular behavior and differentiation in the kidney (65). Calvet et al. (2,66) have proposed two models to explain the immaturity or dedifferentiated state of epithelial cells found in hereditary and acquired cystic kidneys: In infantile cystic disease, the epithelial cells never reach terminal differentiation and are trapped in an immature state, while in adult forms of cystic disease, toxin-induced injury to an initially mature renal epithelium is followed by inability of the epithelium to recover to a fully differentiated state. In genetically cystic *cpk/cpk* mice (a model for autosomal recessive PKD), Deshmukh et al. (53) reported altered levels of ceramide and complex glycosphingolipids in kidney tissue from cystic mice, but not in their phenotypically normal littermates. Lower levels of ceramide and sulfatide, but higher levels of glucosyl- and lactosylceramide and ganglioside GM3, were measured. Our research in infantile and adult human PKD kidney tissue and cyst fluids showed no detectable free sphinganine, the primary sphingoid base formed during de novo biosynthesis of mammalian sphingolipids (54). Thus, anomalies in sphingolipid biology exist in cystic kidney tissue.

It is difficult to separate concepts of sphingolipid biology from considerations of infectious disease, microbial components, and "microsphingoids" in PKD. Potential consequences of alterations in glycosphingolipids on the surface of PKD cells include enhanced microbial

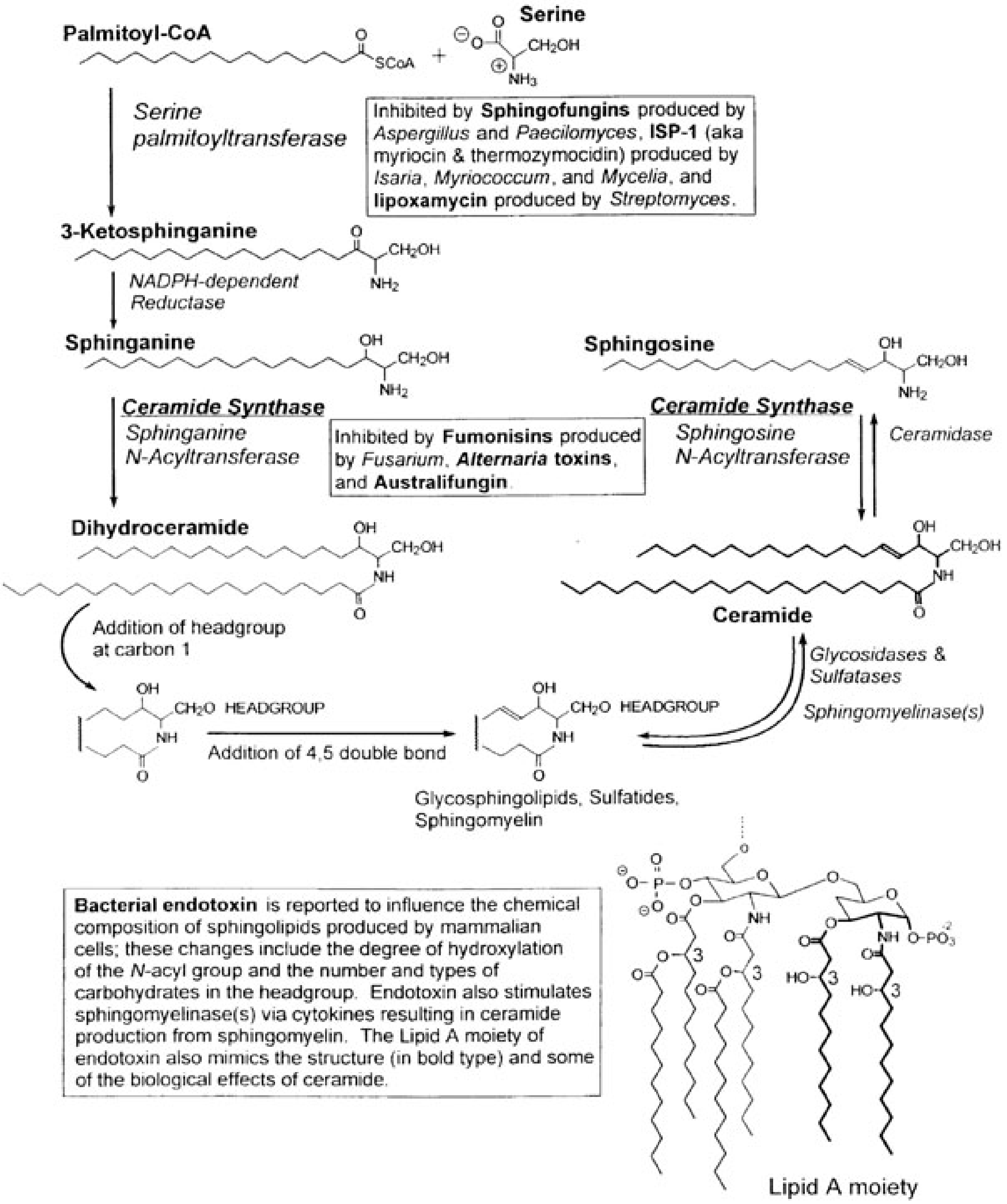

Figure 3. Sites of impact of endotoxin and mycotoxins on sphingolipid metabolism. In PKD, renal sphingolipid formation is altered. Such compromised sphingolipid pathways would be expected to be vulnerable to these highly potent microbial toxins, especially during chronic exposure within renal cysts.

colonization due to the availability of complex sphingolipids as binding sites. Binding of microbes or their components to such sphingolipids may even promote cystogenesis, as binding to complex sphingolipid has been reported to cause changes in differentiation and morphology of cells in vitro (67). Altered biosynthesis of sphingoid bases affects the ratios of sphinganine to sphingosine with consequences to signal transduction (61) and may even alter the antimicrobial environment, as sphingoid bases are reported to have direct antifungal and antibacterial activity (68). The levels of sphingosine in human cyst fluid (Hjelle, unpub.) are comparable to the levels that induce a state of cytoresistance (cellular dedifferentiation) in kidney cells in vitro (69). In addition to products of mammalian sphingolipid metabolism, endotoxin in relatively high amounts was found in cyst fluids from infantile and adult forms of PKD and in simple cysts (Table 1). Could "microsphingoids" or microbial toxins injure and then prevent repair of renal epithelial cells? Data from diverse scientific disciplines support this possibility.

LPS influences nephron formation (70) and renal cystogenesis (11). Fumonisins are potent nephrotoxins (43,55), alter repair mechanisms in kidney cells in vitro (71), and induce apoptosis (72,73). Renal cysts are occasionally reported after chronic exposure of rodents to fumonisins (74). Rates of programmed cell death are abnormal in PKD kidney tissue (75,76); ceramide is a pivotal messenger in apoptosis (64). In PKD, altered processing and sorting of cell membrane proteins and secretory material occurs (2-4); sphingolipids influence processing, sorting, and movement of membranes and ion transporters (64,65), as does fumonisin in kidney cells in vitro (77). In PKD, various electrolyte transport systems are altered (2). cAMP-mediated electrolyte fluxes were proposed as important in cyst formation (3). Fumonisins are reported to activate cAMP response elements (78). Cyst fluid contains uncharacterized substances that promote cystogenesis in vitro (3). Although LPS isolated from *Escherichia coli* did not induce the full array of anticipated cystogenic responses in kidney cells in vitro, the structure, potency, and array of elicited biologic responses of endotoxin depend on the genera and species of bacteria from which it was isolated (13). Coupled with the presence of fungal components, cyst fluid likely represents a complex mixture of "microsphingoids" and toxins that may change over time with dynamic consequences to cyst formation. The heterogeneity of cyst volume and content of growth factors and cytokines in adult PKD has been noted (3,16).

Concepts of infection and "microsphingoids" appear to converge with the expanding knowledge of PKD gene products. The structures of the PKD1 (polycystin) and PKD2 gene products share homology with α1E subtype of voltage-activated, calcium channels (3,6) and a sea urchin protein likely involved in calcium fluxes during fertilization (79). LPS alters voltage-activated, calcium channels (80) and osmo-regulation (81) in mammalian cells. The PKD1 gene product also contains regions of homology with ligand and receptor domains putatively involved in binding to adjacent cells and extra-cellular matrix (3); one such domain is similar to C type lectins that can bind microbes (82). Tissues, including kidney and gut, expressing polycystin in adult PKD, may exhibit a relatively greater leakiness to normal molecular and par-ticulate traffic, as seen in kidney cysts (3) and susceptibility to diverticulosis (3,44). Polycystin also shows homology with apoprotein from low density lipoprotein. Because LPS and sphingo-lipids bind to and are transported by serum lipoproteins, the low-density lipoprotein binding site in polycystin may also enable accumulation and/or transport of "microsphingoids."

Secondary mutations in PKD genes appear to be required for clonal cyst formation (3,5), as is a loss of renal tissue through dysregulation of apoptosis (76). Alterations in the expression of the *bcl*-2 gene product in mice results in polycystic kidneys and dysregulation of apoptosis (83). Although infection increases translocation of the *bcl*-2 gene in human lymphoid tissue (84), it is not known if infection in PKD patients causes such a dysregulation of *bcl*-2 in kidney tissue.

Regarding infection and sphingolipids, ceramide is emerging as a pivotal molecule in the immune system (63) and fumonisins are reported to alter immune function (85). It is ironic that our findings potentially link *Fusarium* to PKD, as fumonisin is used as a molecular tool to study sphingolipid metabolism and signal trandsduction in PKD. Not to be overlooked are the classic bacterial sphingomyelinases encountered during infection and released from gut microflora (e.g., *Staphylococcus aureus* and *Clostridium perfringens*) that can stimulate ceramide formation by hydrolysis of sphingomyelin. Thus, the vulnerability of PKD patients to infection may be related, in part, to anomalies in sphingolipid biology that influence

1) "biostatic" mechanisms of cell regulation, 2) the structure of the plasma membrane and the function of PKD gene products, 3) the availability of glycosphingolipids as binding sites for microbes and their components, 4) the bioavailability of microbial components present in the gut, and 5) antimicrobial defenses in general. Such vulnerability is likely influenced by repeated courses of antimicrobial therapy that provide selection pressure for colonization with a modified microflora. The extent to which sphingolipid biology in PKD is influenced by genetic defects rather than microbial factors is yet to be defined.

Anomalies caused by the PKD gene defect(s) alone cannot explain cystogenesis. As shown by Werder et al. (9), raising genetically cystic mice in a germ-free environment essentially eliminated cystogenesis and increased survivorship to nearly 100% over that of littermates raised in ambient environment for 18 months. Even in cyst fluid from infantile PKD, relatively high levels of endotoxin were found (Table 1). This suggests that microbial toxins are available early in this disease. Prenatal exposures to toxins and "microsphingoids" are unknown. Although our finding of fungal DNA in eight of eight samples of autosomal-dominant PKD tissue and cyst fluids examined suggests an intimate association of fungal exposure to renal cysts, a contributing multifaceted microbial toxicosis involving diet and gut microflora cannot be excluded. By itself, the finding of microbial components at the site of lesion does not prove a causal role for the microbe(s) in disease progression (1). However, a working hypothesis can be formed on the basis of evidence that microbes promote progression of the primary disease and components of the microbes act on mammalian biology to cause effects plausibly related to the known pathophysiology of the disease.

Although there is an established body of knowledge that PKD is a genetic disorder, our data indicate that bacterial endotoxin, ß-DG, and likely other microbial components are available within the kidney to provoke cystogenesis. We have provided chemical and advanced LAL assay-based evidence of bacterial endotoxin and ß-DG in human PKD cyst fluids. From cyst fluids and PKD kidney tissue we have provided evidence of fungal DNA and cell components by serologic testing and PCR with universal fungal primers. We have integrated these findings into a working hypothesis based on emerging knowledge of PKD gene products, altered sphingolipid metabolism in PKD, the effects of LPS on renal cystogenesis, the mimicry of ceramide by LPS and the effects of mycotoxins on mammalian sphingolipid biology and signal transduction, the occurrence of infection in PKD, and the impact of altered gut permeability and microbial colonization on progression of PKD. Is PKD a genetic disease promoted by microbial influences? Tests of this multifaceted hypothesis require awareness of a breadth of issues drawn from numerous scientific disciplines. Identification of the microbes and microbial components involved will require a concerted analysis using highly sensitive and specific methods. As awareness of the importance of sphingolipid biology in health and disease grows, so will the appreciation that microbial influences will need to be considered in pharmacologic studies that seek to manipulate ceramide and complex sphingolipid biochemistry in disease. The ubiquitous and highly potent bacterial endotoxin is again one of the usual suspects examined as provocateur of disease; in this case, endotoxin's modus operandi of coopting the signal transduction machinery of ceramide to cause chronic disease may ultimately be revealed.

### Acknowledgments

This research was supported by grants and gifts from the Methodist Medical Center Foundation; the Children's Miracle Network Telethon; and the Campus Research Board, University of Illinois. We thank Dr. Jim Cooper for his advice, counsel, and support during the endotoxin experiments and preparation of the manuscript; Dr. Kerry O'Donnell for analysis of specimens by PCR for fungal DNA; Dr. Christine Morrison (*Candida* mannan), Dr. Leo Kaufman (*Fusarium* antigen), and Mr. Steven Hurst (*Aspergillus* galactomannan) for fungal serologic tests for antigens; and Dr. Hun-Chi Lin for fungal serologic tests for antibodies. We also thank Jonathan R. Lane and Robert V. Acuff, for the use of, and advice regarding, the gas-chromatograph-mass spectrometer.

Dr. Miller-Hjelle is professor of Microbiology and Diplomate, American Board of Medical Microbiology.

### References

1. Fredricks D, Relman D. Sequence-based identification of microbial pathogens: a reconsideration of Koch's postulates. Clin Microbiol Rev 1996;9:18-33.
2. Martinez RR, Grantham JJ. Polycystic kidney disease: Etiology, pathogenesis, and treatment. Dis Mon 1995;41:698-765.

3. Grantham JJ. The etiology, pathogenesis, and treatment of autosomal dominant polycystic kidney disease: recent advances. Am J Kid Dis 1996;28:788-803.
4. Carone FA, Bacallao R, Kanwar YS. The pathogenesis of polycystic kidney disease. Histol Histophathol 1995;10:213-21.
5. Brasier JL, Henske EP. Loss of the polycystic kidney disease (PKD1) region of chromosome 16p13 in renal cyst cells supports a loss-of-function model for cyst pathogenesis. J Clin Invest. In press.
6. Mochizuki T, Wu G, Hayashi T, Xenophontos SL, Veldhuisen B, Saris JJ, et al. PKD2, a gene for polycystic kidney disease that encodes an integral membrane protein. Science 1996;272:1339-42.
7. Schwab S, Bander S, Saulo K. Renal infection autosomal dominant polycystic kidney disease. Am J Med 1987;82:714-8.
8. Sklar A, Caruana RJ, Lammers JE, Strauser GD. Renal infection in autosomal dominant polycystic kidney disease. Am J Kidney Dis 1987;10:81-8.
9. Werder AA, Amos A, Nielsen AH, Wolfe GH. Comparative effects of germfree and ambient environments on the development of cystic kidney disease in CFWwd mice. J Lab Clin Med 1984;103:399-407.
10. Gardner KD Jr, Evan AP, Reed WP. Accelerated renal cyst development in deconditioned germfree rats. Kidney Int 1986;29:1116-23.
11. Gardner KD Jr, Reed WP, Evan AP, Zedalis J, Hylarides MD, Leon AA. Endotoxin provocation of experimental renal cystic disease. Kidney Int 1987;32:329-34.
12. Munford R, Hall C, Lipton J. Biologic activity, lipoprotein-binding behavior and in vivo disposition of extracted and native forms of *Salmonella typhimurium*. Clin Invest 1982;70:877-88.
13. Rietschel ET, Kirikae T, Schade FU, Mamat U, Schmidt G, Loppnow H, et al. Bacterial endotoxin: molecular relationships of structure to activity and function. FASEB J 1994;8:217-25.
14. Wilkinson SG. Gram-negative bacteria. In: Rutledge G, Wilkinson SG, editors, Microbial Lipids, Vol. 1. New York: Academic Press, 1988:431-57.
15. Miller MA, Prior RB, Horvath FJ, Hjelle JT. Detection of Endotoxiuria in polycystic kidney disease patients by the use of the Limulus Amoebocyte lysate assay. Am J Kid Dis 1990;15:117-22.
16. Gardner KD Jr, Burnside J, Elzinga L. Cytokines in fluids from polycystic kidneys. Kidney Int 1991;39:718-24.
17. Nolan JP. Intestinal endotoxins as mediators of hepatic injury-an idea whose time has come again. Hepatology 1989;10:887-91.
18. Deitch E, Berg R, Specian R. Endotoxin promotes the translocation of bacteria from the gut. Arch Surg 1987;122:185-90.
19. Mimura Y, Yamanaka K, Kawabata R, Inoue A, Sasatomi K, Koga H, et al. Lipopolysaccharide in rheu-matoid arthritis. Journal of Endotoxin Research 1996;3:17.
20. Muhlestein JB, Hammond EH, Carlquist JF, Radicke E, Thomson MJ, Karagounis LA, et al. Increased incidence of Chlamydia species within the coronary arteries of patients with symptomatic atherosclerotic versus other forms of cardiovascular disease. J Am Coll Cardiol 1996;27:1555-61.
21. Raetz CRH, Ulevitch RJ, Wright SD, Sibley CH, Ding A, Nathan CF. Gram negative endotoxin: an extraordinary lipid with profound effects on eukaryotic signal transduction. FASEB J 1991;5:2652-60.
22. Prior RB. The *Limulus* amoebocyte lysate test. In: Prior RB, editor. Clinical applications of the *Limulus* amoebocyte lysate test. Boca Raton (FL): CRC Press; 1990;27-36.
23. Zhang GH, Baei L, Buchardt O, Koch C. Differential blocking of coagulation-activation pathways of *Limulus* amoebocyte lysate. J Clin Microbiol 1994;32:1537-41.
24. Obayashi T, Tamura H, Tanaka S, Ohki M, Takahashi S, Kawai T. Endotoxin-inactivating activity in normal and pathological human blood samples. Infect Immun 1986;53:294-7.
25. Morrison DC, Jacobs DM. Binding of polymyxin B to the lipid A portion of bacterial lipopolysaccharides. Immunochem 1979;13:813-8.
26. Miyazaki T, Hohno S, Mitsutake K, Maesaki S, Tanaka K, Hara K. (1-3)-b-D-glucan in culture fluid of fungi activates Factor G, a *Limulus* coagulation factor. J Clin Lab Anal 1995;9:334-9.
27. Levi ME, Eshaghi N, Smith JW, Elkind C. Fever of unknown origin following an upper gastrointestinal series in a patient with polycystic kidney disease. S Med J 1995;88:769-70.
28. Lowry O, Rosebrough N, Farr A, Randall R. Protein measurement with the Folin phenol reagent. J Biol Chem 1951; 193:265-75.
29. Douwes J, Doekes G, Montijn R, Heedrik D, Brunekreef B. Measurement of b (1-3)-glucan in occu-pational and home environments with an inhibition enzyme immunoassay. Appl Envir Microbiol 1996;62:3176-82.
30. Miyazaki T, Kohno S, Mitsutake K, Yamada H, Yasuoka T, Malsaki S, et al. Combination of conventional and endotoxin-specific Limulus tests for measurement of polysaccharides in sera of rabbits with experimental systemic Candadiasis. Tohoku J Exp Med, 1992;168:1-9.,
31. Sturk A, VanDeventer S, Wortel C. Detection and clinical relevance of human endotoxemia. Zeitschrift fuer Medizinische Laboratoriums diagnostik 1990;31:147-58.
32. Mayberry WR, Lane JR. Sequential alkaline saponification/acid hydrolysis/esterification: a one-tube method with enhanced recovery of both cyclopropane and hydroxylated fatty acids. J Microbiol Methods 1993;18:21-32.
33. Yoshida M, Roth RI, Grunfeld C, Feingold KR, Levin J. Soluble (1-3)-b-D-glucan purified from *Candida albicans*: biologic effects and distribution in blood and organs in rabbits. J Lab Clin Med 1996;128:103-14.
34. Ikemura K, Ikegama K, Shimazu T, Yoshioka T, Sugimoto T. False positive results in Limulus test caused by Limulus amoebocyte lysate-reactive material in immunoglobulin products. J Clin Microbiol 1989;27:1965-8.
35. Williams D. (1-3)-b-D-Glucan. In: Rylander R, Jacobs R, editors. Organic dusts: exposure, effects, and prevention. Boca Raton (FL): Lewis Publishers, 1994;83-5.
36. Kaufman L, Reiss E. Serodiagnosis of fungal diseases. In: Rose N, deMacario E, Fahey J, Friedman H, Penn G, editors. Manual of clinical laboratory immunology. Washington (DC): American Society for Microbiology, 1992:506-28.

37. Innis M, Gelfand D. Optimization of PCRs. In: Innis M, Gelfand D, Sninsky J, White T, editors. PCR protocols- a guide to methods and applications. New York: Academic Press 1990:3-12.
38. Latge J-P, Kobayashi H, Debeaupuis J-P, Diaquin M, Sarfati J, Wieruszeski JM, et al. Chemical and immunological characterization of the extracellular galactomannan of *Aspergillus fumigatus*. Infect Immun 1994;62:5424-33.
39. Notermans S, Dufrenne J, Wijnands L, Engel H. Human serum antibodies to extracellular polysac-charide (EPS) of moulds. J Med Vet Mycol 1988;26:41-8.
40. Garner R, Hudson J. Intravenous injection of Candida-derived mannan results in elevated TNFa levels in serum. Infect Immun 1996;4561-66.
41. Roby R, Sneller M. Incidence of fungal spores at the homes of allergic patients in an agricultural community. II. Correlations of skin test with mold frequency. Ann Allergy Asthma Immunol 1979;43:286-8.
42. De Nus M, Rombouts F, Notermans S. Fusarium molds and their mycotoxins. Journal of Food Safety 1996;16:15-58.
43. Badria FA, Li S, Shier WT. Fumonisins as potential causes of kidney disease. Journal of Toxicology-Toxin Reviews 1996;15:273-92.
44. Scheff R, Zuckerman G, Harter H, Delmez J, Koehler R. Diverticular disease in individuals with chronic renal failure due to polycystic kidney disease. Ann Intern Med 1980;92:202-4.
45. Gans H, Matsumoto K. The escape of endotoxin from the intestine. Surgery, Gynecology and Obstetrics 1974;139:395-402.
46. Grantham JJ. Acquired cystic kidney disease. Kidney Int 1991;40:143-52.
47. Gardner KD Jr, Glew RH, Evan AP, McAteer JA, Bernstein J. Why renal cysts grow. Am J Physiol 1994;266:F353-9.
48. Cook J, Dougherty W, Holt T. Enhanced sensitivity to endotoxin induced by the R-E stimulant, glucan. Circulatory Shock 1980;7:225-38.
49. Rylander R. Endotoxin in the environment. In: Levin J, Alving C, Munford R, Redl H, editors. Bacterial endotoxin: lipopolysaccharides from genes to therapy. New York: Wiley-Liss, Inc. 1995;392:79-90.
50. Miller J. Mycotoxins. In: Rylander R, Jacobs R, editors. Organic dusts: exposure, effects and prevention. Boca Raton (FL): Lewis Pubishers, 1994;87-92.
51. O'Donnell K. Progress towards a phylogenetic classificaton of *Fusarium*. Sydowia 1996;48:57-70.
52. Spiegel S, Merrill AH Jr. Sphingolipid metabolism and cell growth regulation. FASEB J 1996;10:1388-97.
53. Deshmukh GD, Radin NS, Gattone V, Shayman JA. Abnormalities of glycosphingolipid, sulfatide, and ceramide in the polycystic (cpk/cpk) mouse. J Lipid Res 1994;35:1611-21.
54. Hjelle JT, Dombrink-Kurtzman M, Nowak DM, Miller-Hjelle MA, Darras F, Dobbie JW. Ceramide pathways in human polycystic kidney disease. Peritoneal Dialysis International 1996;16:S94.
55. Riley RT, Wang E, Schroeder JJ, Smith ER, Plattner RD, Abbas H. Evidence for disruption of sphingolipid metabolism as a contributing factor in the toxicity and carcinogenicity of fumonisins. Nat Toxins 1996;4:3-15.
56. Merrill AH Jr, Liotta DC, Riley RT. Fumonisins: fungal toxins that shed light on sphingolipid function. Trends in Cell Biology 1996;6:218-23.
57. Merrill AH Jr, Grant AM, Wang E, Bacon CW. Lipids and lipid-like compounds of *Fusarium*. In: Prasad R, Ghannoun MA, editors. Lipids of pathogenic fungi. New York: CRC 1996;199-217.
58. Rutledge G, Wilkinson SG, editors. Microbial lipids, Vol. 1, New York: Academic Press, 1988.
59. Wright SD, Kolesnick RN. Does endotoxin stimulate cells by mimicking ceramide? Immunol Today 1995;16:294-302.
60. Barber SA, Detore G, McNally R, Vogel SN. Stimulation of the ceramide pathway partially mimics lipopolysaccharide-induced responses in murine peritoneal macrophages. Infect Immun 1996;64:3397-400.
61. Ho AK, Peng R, Ho AA, Duffield R, Dombrink-Kurtzman MA. Interactions of fumonisins and sphingoid bases with GTP-binding proteins. Biochemical Archives. In Press.
62. Portner A, Peter-Katalinic J, Brade H, Unland F, Buntemeyer H, Muthing J. Structural characterization of gangliosides from resting and endotoxin-stimulated murine B lymphocytes. Biochemistry 1993;32:12685-93.
63. Ballou LR, Laulederkind SJF, Rosloniec EF, Raghow R. Ceramide signalling and the immune response. Biochim Biophys Acta 1996;1301:273-87.
64. Hannun Y. Functions of ceramide in coordinating cellular responses to stress. Science 1996;274:1855-9.
65. Shayman JA. Sphingolipids: their role in intracellular signaling and renal growth. J Am Soc Nephrol 1996;7:171-82.
66. Calvet JP. Injury and development in polycystic kidney disease. Curr Opin Nephrol Hyperten 1994;3:340-8.
67. Shayman JA, Radin NS. Structure and function of renal glycosphingolipids. Am J Physiol 1991;260:F291-302.
68. Bibel DJ, Aly R, Shah S, Shinefield HR. Sphingosines: antimicrobial barriers of the skin. Acta Derm Venereol Suppl (Stockh) 1993;73:407-11.
69. Iwata M, Herrington J, Zager RA. Sphingosine: a mediator of acute renal tubular injury and subsequent cytoresistance. Proc Natl Acad Sci USA 1995;92:8970-4.
70. Woolf AS, Neuhaus TJ, Kolatsi M, Winyard PJ, Klein NJ. Nephron formation is inhibited by lipopolysaccaride and by tumor necrosis factor-a. J Am Soc Nephrol 1994;5:641.
71. Counts RS, Nowak G, Wyatt RD, Schnellmann RG. Nephrotoxicant inhibition of renal proximal tubule cell regeneration. Am J Physiol 1995;269:F274-81.
72. Lim CW, Parker HM, Vesonder RF, Haschek WM. Intravenous fumonisin $B_1$ induces cell proliferation and apoptosis in the rat. Nat Toxins 1996;4:34-41.
73. Wang W, Jones C, Ciacci-Zanella J, Holt T, Gilchrist DG, Dickman MB. Fumonisins and *Alternaria alternata lycopersici* toxins: sphinganine analog mycotoxins induce apoptosis in monkey kidney cells. Proc Natl Acad Sci USA 1996;93:3461-5.
74. Gelderblom WCA, Kriek NPJ, Marasas WFO, Thiel PG. Toxicity and carcinogenicity of the *Fusarium moniliforme* metabolite, fumonisin $B_1$. Carcinogenesis 1991;12:1247-51.

75. Woo D. Apoptosis and loss of renal tissue in polycystic kidney disease. New Eng J Med 1995;333:18-25.
76. Winyard PJD, Nauta J, Lerienman DS, Hardman P, Sams VR, Risdon RA, et al. Deregulation of cell survival in cystic and dysplastic renal development. Kidney Int 1996;49:135-46.
77. Mays RW, Siemers KA, Fritz BA, Lowe AW, van Meer G, Nelson WJ. Hierarchy of mechanisms involved in generating Na/K-ATPase polarity in MDCK epithelial cells. J Cell Biol 1995;130:1105-15.
78. Huang C, Dickman M, Henderson G, Jones C. Repres-sion of protein kinase C and stimulation of cyclic AMP response elements by fumonisin, a fungal encoded toxin which is a carcinogen. Cancer Res 1995;55:1655-9.
79. Moy GW, Mendoza LM, Schulz JR, Swanson WJ, Glabe CG, Vacquier VD. The sea urchin sperm receptor for egg jelly is a modular protein with extensive homology to the human polycystic kidney disease protein, PKD1. J Cell Biol 1996;133:809-17.
80. Wilkinson MF, Earle ML, Triggle CR, Barnes S. Interleukin-1b, tumor necrosis factor-a, and LPS enhance calcium channel current in isolated vascular smooth muscle cells of rat tail artery. FASEB J 1996;10:785-91.
81. Han J, Lee J-D, Bibbs L, Ulevitch RJ. A MAP kinase targeted by endotoxin and hyperosmolarity in mammalian cells. Science 1994;265:808-11.
82. Malhotra R. Collectin receptor (C1q receptor): structure and function. Behring Inst Mitt 1993;93:254-61.
83. Veis DJ, Sorenson CM, Shutter JR, Korsmeyer SJ. Bcl-2-deficient mice demonstrate fulminant lympoid apoptosis, polycystic kidneys, and hypopigmented hair. Cell 1993;75:229-40.
84. Reed JC. Bcl-2 and the regulation of programmed cell death. J Cell Biol 1994;124:1-6.
85. Martinova EA, Merrill AH Jr. Fumonisin $B_1$ alters sphingolipid metabolism and immune function in BALB/c mice: immunologicial responses to fumonisin $B_1$. Mycopathologica 1995;130:163-70.

## References

[PDF_01319] Ballou L. R., Laulederkind S. J., Rosloniec E. F., Raghow R. (1996). Ceramide signalling and the immune response.. Biochim Biophys Acta.

[PDF_01307] Barber S. A., Detore G., McNally R., Vogel S. N. (1996). Stimulation of the ceramide pathway partially mimics lipopolysaccharide-induced responses in murine peritoneal macrophages.. Infect Immun.

[PDF_01331] Bibel D. J., Aly R., Shah S., Shinefield H. R. (1993). Sphingosines: antimicrobial barriers of the skin.. Acta Derm Venereol.

[PDF_01327] Calvet J. P. (1994). Injury and development in polycystic kidney disease.. Curr Opin Nephrol Hypertens.

[PDF_01108] Carone F. A., Bacallao R., Kanwar Y. S. (1995). The pathogenesis of polycystic kidney disease.. Histol Histopathol.

[PDF_01269] Cook J. A., Dougherty W. J., Holt T. M. (1980). Enhanced sensitivity to endotoxin induced by the RE stimulant, glucan.. Circ Shock.

[PDF_01341] Counts R. S., Nowak G., Wyatt R. D., Schnellmann R. G. (1995). Nephrotoxicant inhibition of renal proximal tubule cell regeneration.. Am J Physiol.

[PDF_01155] Deitch E. A., Berg R., Specian R. (1987). Endotoxin promotes the translocation of bacteria from the gut.. Arch Surg.

[PDF_01283] Deshmukh G. D., Radin N. S., Gattone V. H., Shayman J. A. (1994). Abnormalities of glycosphingolipid, sulfatide, and ceramide in the polycystic (cpk/cpk) mouse.. J Lipid Res.

[PDF_01196] Douwes J., Doekes G., Montijn R., Heederik D., Brunekreef B. (1996). Measurement of beta(1-->3)-glucans in occupational and home environments with an inhibition enzyme immunoassay.. Appl Environ Microbiol.

[PDF_01097] Fredricks D. N., Relman D. A. (1996). Sequence-based identification of microbial pathogens: a reconsideration of Koch's postulates.. Clin Microbiol Rev.

[PDF_01261] Gans H., Matsumoto K. (1974). The escape of endotoxin from the intestine.. Surg Gynecol Obstet.

[PDF_01150] Gardner K. D., Burnside J. S., Elzinga L. W., Locksley R. M. (1991). Cytokines in fluids from polycystic kidneys.. Kidney Int.

[PDF_01129] Gardner K. D., Evan A. P., Reed W. P. (1986). Accelerated renal cyst development in deconditioned germ-free rats.. Kidney Int.

[PDF_01266] Gardner K. D., Glew R. H., Evan A. P., McAteer J. A., Bernstein J. (1994). Why renal cysts grow.. Am J Physiol.

[PDF_01132] Gardner K. D., Reed W. P., Evan A. P., Zedalis J., Hylarides M. D., Leon A. A. (1987). Endotoxin provocation of experimental renal cystic disease.. Kidney Int.

[PDF_01244] Garner R. E., Hudson J. A. (1996). Intravenous injection of Candida-derived mannan results in elevated tumor necrosis factor alpha levels in serum.. Infect Immun.

[PDF_01353] Gelderblom W. C., Kriek N. P., Marasas W. F., Thiel P. G. (1991). Toxicity and carcinogenicity of the Fusarium moniliforme metabolite, fumonisin B1, in rats.. Carcinogenesis.

[PDF_01264] Grantham J. J. (1991). Acquired cystic kidney disease.. Kidney Int.

[PDF_01105] Grantham J. J. (1996). The etiology, pathogenesis, and treatment of autosomal dominant polycystic kidney disease: recent advances.. Am J Kidney Dis.

[PDF_01383] Han J., Lee J. D., Bibbs L., Ulevitch R. J. (1994). A MAP kinase targeted by endotoxin and hyperosmolarity in mammalian cells.. Science.

[PDF_01322] Hannun Y. A. (1996). Functions of ceramide in coordinating cellular responses to stress.. Science.

[PDF_01370] Huang C., Dickman M., Henderson G., Jones C. (1995). Repression of protein kinase C and stimulation of cyclic AMP response elements by fumonisin, a fungal encoded toxin which is a carcinogen.. Cancer Res.

[PDF_01218] Ikemura K., Ikegami K., Shimazu T., Yoshioka T., Sugimoto T. (1989). False-positive result in Limulus test caused by Limulus amebocyte lysate-reactive material in immunoglobulin products.. J Clin Microbiol.

[PDF_01334] Iwata M., Herrington J., Zager R. A. (1995). Sphingosine: a mediator of acute renal tubular injury and subsequent cytoresistance.. Proc Natl Acad Sci U S A.

[PDF_01193] LOWRY O. H., ROSEBROUGH N. J., FARR A. L., RANDALL R. J. (1951). Protein measurement with the Folin phenol reagent.. J Biol Chem.

[PDF_01236] Latgé J. P., Kobayashi H., Debeaupuis J. P., Diaquin M., Sarfati J., Wieruszeski J. M., Parra E., Bouchara J. P., Fournet B. (1994). Chemical and immunological characterization of the extracellular galactomannan of Aspergillus fumigatus.. Infect Immun.

[PDF_01189] Levi M. E., Eshaghi N., Smith J. W., Elkind C. (1995). Fever of unknown origin following an upper gastrointestinal series in a patient with polycystic kidney disease.. South Med J.

[PDF_01344] Lim C. W., Parker H. M., Vesonder R. F., Haschek W. M. (1996). Intravenous fumonisin B1 induces cell proliferation and apoptosis in the rat.. Nat Toxins.

[PDF_01386] Malhotra R. (1993). Collectin receptor (C1q receptor): structure and function.. Behring Inst Mitt.

[PDF_01100] Martinez J. R., Grantham J. J. (1995). Polycystic kidney disease: etiology, pathogenesis, and treatment.. Dis Mon.

[PDF_01394] Martinova E. A., Merrill A. H. (1995). Fumonisin B1 alters sphingolipid metabolism and immune function in BALB/c mice: immunological responses to fumonisin B1.. Mycopathologia.

[PDF_01366] Mays R. W., Siemers K. A., Fritz B. A., Lowe A. W., van Meer G., Nelson W. J. (1995). Hierarchy of mechanisms involved in generating Na/K-ATPase polarity in MDCK epithelial cells.. J Cell Biol.

[PDF_01295] Merrill A. H., Liotta D. C., Riley R. T. (1996). Fumonisins: fungal toxins that shed light on sphingolipid function.. Trends Cell Biol.

[PDF_01146] Miller M. A., Prior R. B., Horvath F. J., Hjelle J. T. (1990). Detection of endotoxiuria in polycystic kidney disease patients by the use of the Limulus amebocyte lysate assay.. Am J Kidney Dis.

[PDF_01185] Miyazaki T., Kohno S., Mitsutake K., Maesaki S., Tanaka K., Hara K. (1995). (1-->3)-beta-D-glucan in culture fluid of fungi activates factor G, a limulus coagulation factor.. J Clin Lab Anal.

[PDF_01115] Mochizuki T., Wu G., Hayashi T., Xenophontos S. L., Veldhuisen B., Saris J. J., Reynolds D. M., Cai Y., Gabow P. A., Pierides A. (1996). PKD2, a gene for polycystic kidney disease that encodes an integral membrane protein.. Science.

[PDF_01374] Moy G. W., Mendoza L. M., Schulz J. R., Swanson W. J., Glabe C. G., Vacquier V. D. (1996). The sea urchin sperm receptor for egg jelly is a modular protein with extensive homology to the human polycystic kidney disease protein, PKD1.. J Cell Biol.

[PDF_01161] Muhlestein J. B., Hammond E. H., Carlquist J. F., Radicke E., Thomson M. J., Karagounis L. A., Woods M. L., Anderson J. L. (1996). Increased incidence of Chlamydia species within the coronary arteries of patients with symptomatic atherosclerotic versus other forms of cardiovascular disease.. J Am Coll Cardiol.

[PDF_01135] Munford R. S., Hall C. L., Lipton J. M., Dietschy J. M. (1982). Biological activity, lipoprotein-binding behavior, and in vivo disposition of extracted and native forms of Salmonella typhimurium lipopolysaccharides.. J Clin Invest.

[PDF_01152] Nolan J. P. (1989). Intestinal endotoxins as mediators of hepatic injury--an idea whose time has come again.. Hepatology.

[PDF_01241] Notermans S., Dufrenne J., Wijnands L. M., Engel H. W. (1988). Human serum antibodies to extracellular polysaccharides (EPS) of moulds.. J Med Vet Mycol.

[PDF_01178] Obayashi T., Tamura H., Tanaka S., Ohki M., Takahashi S., Kawai T. (1986). Endotoxin-inactivating activity in normal and pathological human blood samples.. Infect Immun.

[PDF_01315] Pörtner A., Peter-Katalinić J., Brade H., Unland F., Büntemeyer H., Müthing J. (1993). Structural characterization of gangliosides from resting and endotoxin-stimulated murine B lymphocytes.. Biochemistry.

[PDF_01167] Raetz C. R., Ulevitch R. J., Wright S. D., Sibley C. H., Ding A., Nathan C. F. (1991). Gram-negative endotoxin: an extraordinary lipid with profound effects on eukaryotic signal transduction.. FASEB J.

[PDF_01392] Reed J. C. (1994). Bcl-2 and the regulation of programmed cell death.. J Cell Biol.

[PDF_01139] Rietschel E. T., Kirikae T., Schade F. U., Mamat U., Schmidt G., Loppnow H., Ulmer A. J., Zähringer U., Seydel U., Di Padova F. (1994). Bacterial endotoxin: molecular relationships of structure to activity and function.. FASEB J.

[PDF_01291] Riley R. T., Wang E., Schroeder J. J., Smith E. R., Plattner R. D., Abbas H., Yoo H. S., Merrill A. H. (1996). Evidence for disruption of sphingolipid metabolism as a contributing factor in the toxicity and carcinogenicity of fumonisins.. Nat Toxins.

[PDF_01247] Roby R. R., Sneller M. R. (1979). Incidence of fungal spores at the homes of allergic patients in an agricultural community. II. Correlations of skin tests with mold frequency.. Ann Allergy.

[PDF_01257] Scheff R. T., Zuckerman G., Harter H., Delmez J., Koehler R. (1980). Diverticular disease in patients with chronic renal failure due to polycystic kidney disease.. Ann Intern Med.

[PDF_01119] Schwab S. J., Bander S. J., Klahr S. (1987). Renal infection in autosomal dominant polycystic kidney disease.. Am J Med.

[PDF_01329] Shayman J. A., Radin N. S. (1991). Structure and function of renal glycosphingolipids.. Am J Physiol.

[PDF_01324] Shayman J. A. (1996). Sphingolipids: their role in intracellular signaling and renal growth.. J Am Soc Nephrol.

[PDF_01122] Sklar A. H., Caruana R. J., Lammers J. E., Strauser G. D. (1987). Renal infections in autosomal dominant polycystic kidney disease.. Am J Kidney Dis.

[PDF_01281] Spiegel S., Merrill A. H. (1996). Sphingolipid metabolism and cell growth regulation.. FASEB J.

[PDF_01206] Sturk A., van Deventer S. J., Wortel C. H., Levels J. H., ten Cate J. W., Büller H. R., Sanders G. T. (1990). Detection and clinical relevance of human endotoxemia.. Z Med Lab Diagn.

[PDF_01388] Veis D. J., Sorenson C. M., Shutter J. R., Korsmeyer S. J. (1993). Bcl-2-deficient mice demonstrate fulminant lymphoid apoptosis, polycystic kidneys, and hypopigmented hair.. Cell.

[PDF_01348] Wang W., Jones C., Ciacci-Zanella J., Holt T., Gilchrist D. G., Dickman M. B. (1996). Fumonisins and Alternaria alternata lycopersici toxins: sphinganine analog mycotoxins induce apoptosis in monkey kidney cells.. Proc Natl Acad Sci U S A.

[PDF_01125] Werder A. A., Amos M. A., Nielsen A. H., Wolfe G. H. (1984). Comparative effects of germfree and ambient environments on the development of cystic kidney disease in CFWwd mice.. J Lab Clin Med.

[PDF_01379] Wilkinson M. F., Earle M. L., Triggle C. R., Barnes S. (1996). Interleukin-1beta, tumor necrosis factor-alpha, and LPS enhance calcium channel current in isolated vascular smooth muscle cells of rat tail artery.. FASEB J.

[PDF_01362] Winyard P. J., Nauta J., Lirenman D. S., Hardman P., Sams V. R., Risdon R. A., Woolf A. S. (1996). Deregulation of cell survival in cystic and dysplastic renal development.. Kidney Int.

[PDF_01360] Woo D. (1995). Apoptosis and loss of renal tissue in polycystic kidney diseases.. N Engl J Med.

[PDF_01304] Wright S. D., Kolesnick R. N. (1995). Does endotoxin stimulate cells by mimicking ceramide?. Immunol Today.

[PDF_01214] Yoshida M., Roth R. I., Grunfeld C., Feingold K. R., Levin J. (1996). Soluble (1-->3)-beta-D-glucan purified from Candida albicans: biologic effects and distribution in blood and organs in rabbits.. J Lab Clin Med.

[PDF_01175] Zhang G. H., Baek L., Buchardt O., Koch C. (1994). Differential blocking of coagulation-activating pathways of Limulus amebocyte lysate.. J Clin Microbiol.

